# Sustained Acoustic Medicine Combined with A Diclofenac Ultrasound Coupling Patch for the Rapid Symptomatic Relief of Knee Osteoarthritis: Multi-Site Clinical Efficacy Study

**DOI:** 10.2174/1874325002014010176

**Published:** 2020-12-18

**Authors:** Alex Madzia, Chirag Agrawal, Paddy Jarit, Stephanie Petterson, Kevin Plancher, Ralph Ortiz

**Affiliations:** 1 Department of Biomedical Engineering, University of Cincinnati, Cincinnati, OH 45219, USA; 2 Sport and Orthopaedic Physical Therapy, Fairfield, CT 06824, USA; 3 The Orthopaedic Foundation, Stamford, CT 06824, USA; 4 Albert Einstein College of Medicine, Bronx, NY, New York, USA; 5 Weill Cornell Medical College, New York, NY 13053, USA; 6 Plancher Orthopaedics & Sports Medicine, New York, NY 13053, USA; 7 Medical Pain Consultants, Dryden, NY 13053, USA

**Keywords:** Sustained acoustic medicine, Sonophoresis, Diclofenac, Anti-steroidal anti-inflammatory drugs, Long-duration continuous ultrasound, Low-intensity continuous ultrasound, Pain management

## Abstract

**Background:**

Sustained Acoustic Medicine (SAM) is an emerging, non-invasive, non-narcotic, home-use ultrasound therapy for the daily treatment of joint pain. The aim of this multi-site clinical study was to examine the efficacy of long-duration continuous ultrasound combined with a 1% diclofenac ultrasound gel patch in treating pain and improving function in patients with knee osteoarthritis.

**Methods:**

The Consolidated Standards of Reporting Trials (CONSORT) were followed. Thirty-two (32) patients (18-males, 14-females) 54 years of average age with moderate to severe knee pain and radiographically confirmed knee osteoarthritis (Kellgren-Lawrence (KL) grade II/III) were enrolled for treatment with the SAM device and diclofenac patch applied daily to the treated knee. SAM ultrasound (3 MHz, 0.132 W/cm^2^, 1.3 W) and 6 grams of 1% diclofenac were applied with a wearable device for 4 hours daily for 1 week, delivering 18,720 Joules of ultrasound energy per treatment. The primary outcome was the daily change in pain intensity using a numeric rating scale (NRS 0–10), which was assessed prior to intervention (baseline, day 1), before and after each daily treatment, and after 1 week of daily treatment (day 7). Rapid responders were classified as those patients exhibiting greater than a 1-point reduction in pain following the first treatment. Change in Western Ontario McMaster Osteoarthritis Questionnaire (WOMAC) score from baseline to day 7 was the secondary functional outcome measure. Additionally, a series of daily usability and user experience questions related to devising ease of use, functionality, safety, and effectiveness, were collected. Data were analyzed using t-tests and repeated measure ANOVAs.

**Results:**

The study had a 94% retention rate, and there were no adverse events or study-related complaints across 224 unique treatment sessions. Rapid responders included 75% of the study population. Patients exhibited a significant mean NRS pain reduction over the 7-day study of 2.06-points (50%) for all subjects (n=32, p<0.001) and 2.96-points (70%) for rapid responders (n=24, p<0.001). The WOMAC functional score significantly improved by 351 points for all subjects (n=32, p<0.001), and 510 points for rapid responders (n=24, p<0.001). Over 95% of patients found the device safe, effective and easy to use, and would continue treatment for their knee OA symptoms.

**Conclusion:**

Sustained Acoustic Medicine combined with 1% topical diclofenac rapidly reduced pain and improved function in patients with moderate to severe osteoarthritis-related knee pain. The clinical findings suggest that this treatment approach may be used as a conservative, non-invasive treatment option for patients with knee osteoarthritis. Additional research is warranted on non-weight bearing joints of the musculoskeletal system as well as different topical drugs that could benefit from improved localized delivery.

Clinical Trial Registry Number: (NCT04391842).

## 1. INTRODUCTION

Osteoarthritis (OA) affects 37.4% of adults in the United States over the age of 60, costing the American economy approximately $60 billion per year [1]. OA development is related to genetic factors, aging, obesity, and joint misalignment [2 – 5]. Progression of OA is associated with chronic pain, joint instability, stiffness, narrowing, and joint degeneration [3 – 7]. While the underlying mechanism of OA progression and associated pain is not well understood, inflammation plays an important role in the degradation of affected joints over time. Treatment of OA is limited to pain management, reducing joint stiffness, improving range of motion, function, and the patient’s quality of life living with the disease [8]. Current symptomatic treatment of OA includes weight loss, exercise, physical therapy, acupuncture, therapeutic ultrasound, hyaluronic acid injection, systemic or topical application of nonsteroidal anti-inflammatory drugs (NSAIDs), and surgical intervention. Many of these treatments provide transient pain relief, break the integrity of the skin, and have long term adverse side effects or procedural risk [8 – 15].

Nonsteroidal anti-inflammatory drugs (NSAIDs) are one of the most common treatments of OA and have been used to manage OA associated pain for decades. Long-term use of systemic NSAIDs have adverse effects on the kidney, liver, and gastrointestinal system [9, 16 – 18], while the topical application of NSAIDs has limited penetration through the skin [9, 19]. Recently, multiple meta-analyses have shown higher efficacy of diclofenac relative to other NSAIDs such as ibuprofen, celecoxib, and ketoprofen [20, 21]. Multiple studies have used different methods to increase the efficacy of diclofenac penetration through the skin, such as using skin penetration enhancers, electroporation, iontophoresis, and sonophoresis [22 – 25].

Ultrasound has been considered a potential therapy to alleviate pain associated with OA progression [26 – 29]. The effectiveness of ultrasound therapy is highly dependent on multiple parameters such as intensity, duty cycle, frequency, duration of application, and energy dose [30, 31]. Sustained Acoustic Medicine (SAM) has emerged in the last decade as a well-controlled, high-dose, home-use ultrasound treatment for patients with musculoskeletal injuries [32 – 35]. Multiple meta-analyses have shown that ultrasound with correct dosing can be used as stand-alone or adjunctive therapy to manage OA associated pain [29, 36 – 38]. Huang *et al.* (2005) showed the effectiveness of ultrasound (25% duty, 1 MHz, 2.5 W/cm^2^) as adjunct OA therapy improving physical activity. Patients showed an increase in knee range of motion (ROM) and a decrease in pain on the visual analog scale (VAS) after 8 weeks of treatment [27].

Similarly, Yang *et al.* (2006) reported a significant increase in VAS scores after 28 days for ultrasound treatment for OA [39]. In two series (n=12, n=7) of SAM wearable, long-duration, continuous ultrasound studies on knee OA, Langer *et al.* (2014) reported that daily SAM treatment for 12 to 60 days significantly reduced pain by 52% (2 to 4 -points improvement on the VAS), and demonstrated a 20% improvement in joint function for the active versus placebo treatment group. The authors concluded that the SAM studies were underpowered for the determination of clinical significance and that a larger clinical trial was warranted [40]. Langer *et al.* (2015) reported in a 47 patient, randomized placebo-controlled study (28 active and 19 placebo) on SAM treatment of radiographic mild to moderate clinical knee OA (Grade 1–2 on the Osteoarthritis Research Society International (OARSI) scale), and demonstrated that patients with moderate to severe pain had a 2.5-point reduction in pain on the VAS over 42 days of treatment which was statistically significant over the placebo 1.23-point decrease. The authors concluded that reduction in OA pain was clinically meaningful and exceeded *The Initiative on Methods, Measurements, and Pain Assessment in Clinical Trials* (IMMPACT) guidelines [34]. In a double-blind, randomized, placebo-controlled clinical trial on SAM treatment for knee OA by Draper *et al.* (2018), 93 patients with moderate to severe pain and mild radiographic knee OA (Kellgren-Lawrence grade I/II) had a significant 1.96-point pain reduction for active versus 0.85 point reduction for placebo on a numeric rating scale (NRS). Patients receiving active SAM treatment also had significant improvement in pain, stiffness, and function on the Western Ontario and McMaster Universities (WOMAC) scale compared with placebo (500 vs. 311, respectively). The authors concluded that SAM treatment significantly reduced pain and improved joint function in patients with moderate to severe OA knee pain and could be used as a conservative treatment option for patients with knee OA [33]. A recent 2019 systematic review and meta-analysis on ultrasound dosing for knee OA demonstrated that regular ultrasound treatment significantly relieved pain and reduced the WOMAC physical function score. Ultrasound also increased the active range of motion and reduced the Lequesne index. The authors concluded that regular ultrasound treatment is safe and effective at relieving pain and improving physical function in patients with knee OA [37, 41]. The objective of this study was to determine the efficacy of long-duration continuous ultrasound delivered by the SAM device combined with 1% diclofenac for the symptomatic treatment of knee OA pain. We hypothesized that combining SAM with topical diclofenac would provide more rapid pain reduction for patients compared with prior SAM studies reported in the clinical literature.

## 2. METHODS

The study and methods followed the Consolidated Standards of Reporting Trials (CONSORT) [42]. The prospective multi-site study was conducted in the Central New York and Southern Coastal Connecticut regions of the United States between June 2019 and January 2020 registered with ClinicalTrials.gov identifier NCT04391842. Patient enrollment was accomplished through referrals from Cayuga Medical Center and the Yale-New Haven Health System community hospitals, to Medical Pain Consultants and Sport and Orthopaedic Physical Therapy, affiliated outpatient care practices, respectively. The practices served as the setting for enrollment, training on the use of the device, visits of the patients with research staff, and pre/post functional measurements. The patient’s home/work setting served as the setting at which the device was self-administered and where pain measurements were recorded. The study was approved by the institutional review board of the Integ Review, and all patients provided informed consent to participate. The procedures followed were in accordance with the ethical standards of the responsible committee on human experimentation and with the Helsinki Declaration of 1975, as revised in 2000 [43].

Included patients were 45 to 85 years of age, reported moderate to severe knee OA pain negatively affecting their life, were radiologically confirmed to have mild to moderate OA (Kellgren-Lawrence (KL) grade II-III score) in one or both knees, had baseline day-1 pain Numerical Rate Score (NRS 0–10) pain between 3 and 7, had no intraarticular injection to the treated knee in the last 6 months, had no trauma to the treated knee, and had no implants or surgeries to the treated knee. In cases of bilateral knee OA, the more painful knee was selected for treatment; if equal pain, a flip of a coin was used to select the knee for treatment. Participants were excluded if they had KL score greater than III, showed an inability to apply the device, were currently using a steroid-based medication, had a recent history of trauma to the knee or having osteoarthritis develop secondary to a metabolic disorder.

**Fig. (1).**
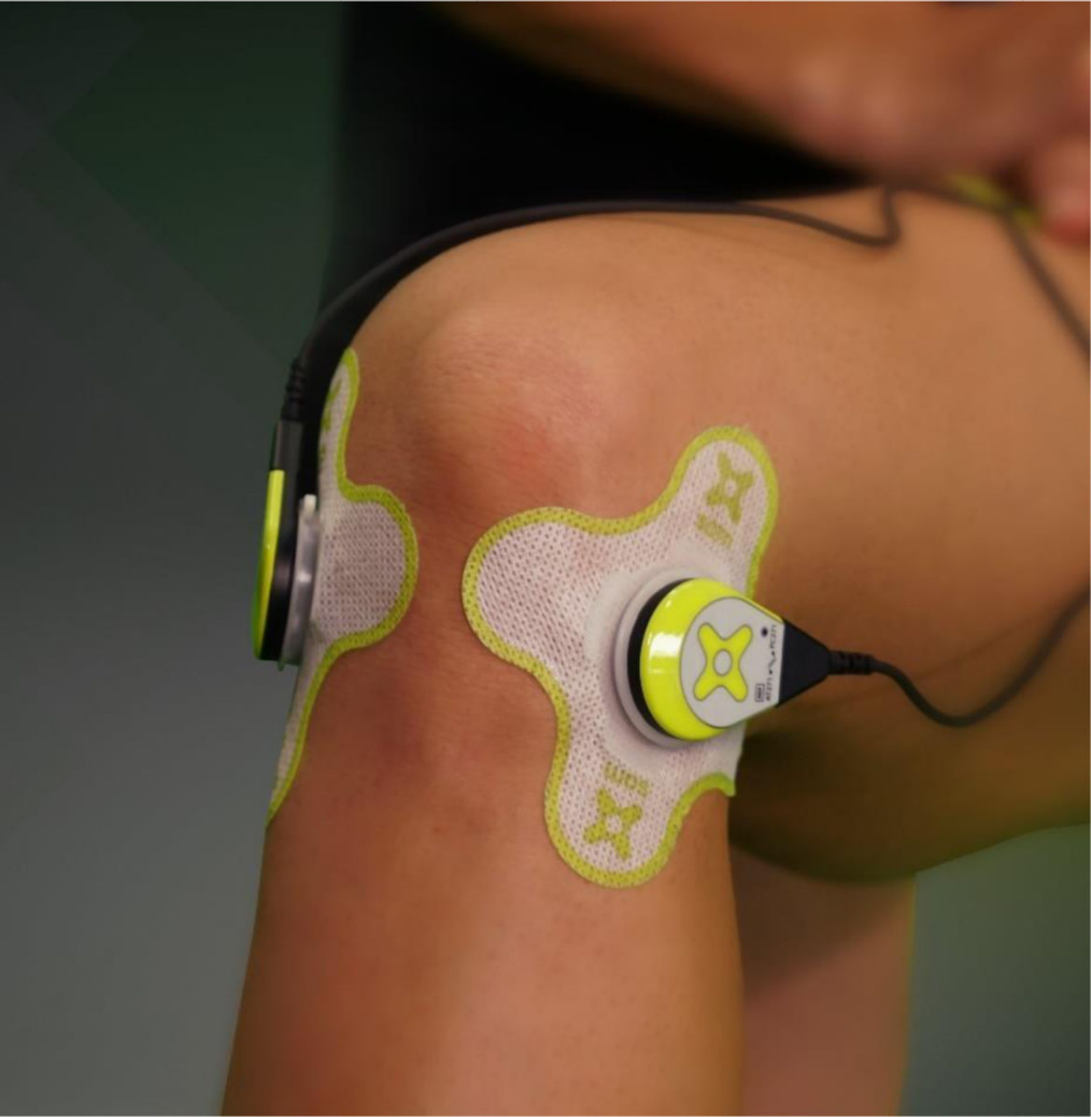
Sustained Acoustic Medicine (SAM) ultrasound device applied to the medial and lateral articulation points of the knee with 1% diclofenac ultrasound coupling patch.

Patients meeting inclusion criteria were enrolled for a 7-day treatment with SAM ultrasound device and 1% diclofenac ultrasound coupling patch. Patients subsequently self-applied the respective treatment 4 hours per day for 1 week to the lateral and medial arthritic knee, as shown in Fig. (1). Measurements of pain before and after daily application of SAM and ease of use were recorded in a daily patient diary, while functional measurements were completed during clinic visits.

The clinical sample size for the study was determined from Draper *et al.* (2018) using the mean knee OA pain reduction from SAM treatment for the first 2 weeks of the study (active mean 3.61 ± 2.53) and mean baseline pain of the study group (mean 5.53 ± 2.37); A sample size of 23 patients provided over 95% power for the primary outcome measure NRS pain reduction. We conservatively targeted enrollment of 30 patients anticipating insignificant (less than 5%) dropouts for the 1-week study. We also anticipated a stronger treatment effect size for pain reduction in our study since SAM was to be applied for 4-hours with topical diclofenac per treatment versus only SAM in Draper *et al.* (2018). Total participants completing the study was slightly above target (+2).

### 2.1. Baseline Measurements and Intervention Protocol

The patients’ initial NRS and Western Ontario and McMaster Universities Arthritis Index (WOMAC) scores were recorded at the outpatient care facilities on Day 1 of the study. Patients were trained to use the Sustained Acoustic Medicine (SAM) device along with diclofenac patch and record NRS pain scores before the treatment and post 4-hour treatment in daily diaries for 7 days. SAM® Pro 2.0 (ZetrOZ Systems LLC, Trumbull, CT) is a portable, wearable, US Food and Drug Administration FDA-approved Class II medical device for prescription home-use ultrasound treatment. The SAM device delivers ultrasound at 3 MHz, 100% duty cycle, 1.3 W with 132 mw/cm^2^ intensity per transducer and overall energy delivery of 18,720 Joules over 4 hours of treatment. The device is attached to the body with a disposable adhesive patch which was pre-filled with 3g ultrasonic coupling gel with 1% diclofenac provided by the manufacturer (Fig. 1).

At the clinics, patients were shown how to apply the disposable adhesive patches/transducers to the medial and lateral sides of the arthritic knee and set the medical devices treatment timer for 4 hours of continuous ultrasound (Fig. 1). Patients were instructed to wear the device during regular daily activity and apply/remove the device when convenient with their daily schedule. Each patient received one rechargeable device and 18 disposable, single use, diclofenac ultrasound gel adhesive patches. Patients were instructed to apply the device daily and record pain and usability questions daily in the diary.

Patients recorded the primary outcome NRS (0 – 10, 0 = no pain, 10 = extreme pain) at pre-treatment and post-treatment over 7 days. The secondary outcome WOMAC was recorded at the outpatient care practices at the beginning of the study and after 7 days of treatment, evaluating activity, stiffness, and function of the treated knee.

### 2.2. Follow-up and Statistical Analysis

Once enrolled in the study, patients completed outpatient care facility visits on day 1 (patient screening, enrollment and informed consent) and day 7 (study completion). During the week, the research staff talked with the patient once on the phone to review the daily pain diary, addressed any questions the patient had about using the device or being involved in the study, and monitored for any adverse events (*i.e.*, a serious unanticipated injury or death) or reactions (*e.g.*, skin sensitivity, redness or burn) from the device.

Change in NRS pain score from baseline was analyzed for pre and post-treatment each treatment day throughout the study, and WOMAC (pain, stiffness, and functional change) was evaluated on day 1 and day 7. Demographic and outcomes data were analyzed using t-tests and repeated measure ANOVAs. Based on the primary outcome measure, rapid responders were considered to have greater than a 1-point reduction in pain on the first treatment. Chi-squared proportional assessment was used to assess gender demographics between groups. Data analysis was conducted in the R software environment for statistical computing (The R Foundation for Statistical Computing, Vienna, Austria). Data are expressed as means ± SDs (standard deviations). The p-values of less than 0.05 were considered statistically significant.

## 3. RESULTS

**Fig. (2).**
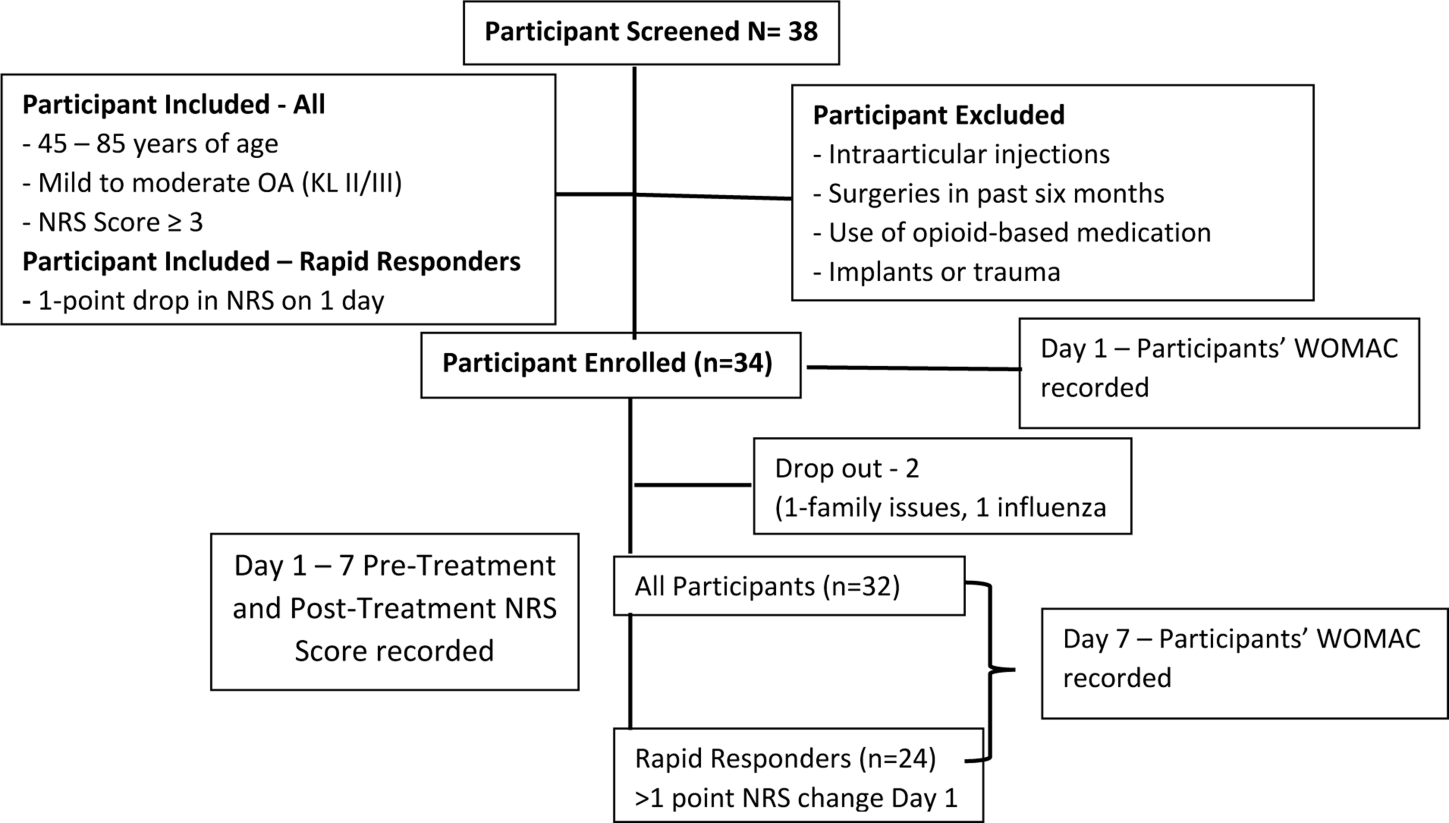
Flow chart illustrating study design: Participants screening, exclusion criteria, and data collection time points.

A total of 38 patients were screened. Thirty-four [33] patients were eligible and enrolled in the study. Thirty-two [31] completed the study. The two dropouts were non-study related on the first day of the protocol (one due to family and one due to influenza), resulting in a 94% retention rate. A total of 18 males and 14 females completed the 7-day study (Fig. 2). The patient demographics for treatment intervention include subjects with mean age 53.6 ± 8.5 years and body mass index (BMI) 32.8 ± 8.8. Patients reported moderate knee OA pain at baseline, average NRS 4.06± 2.39. No significant differences or trends were found between baseline pain and BMI by gender. Approximately 62% (20 patients) of the study population were non-Hispanic Caucasian and 38% (12 patients) non-Hispanic African American. Enrolled patients were currently seeing medical care for knee osteoarthritis pain. The most common pain medications were prescription NSAIDs and oxycodone. The most common non-drug pain treatment was physical therapy and light exercise. Cointervention results were not investigated in this study.

### 3.1. Primary Outcome Measure of Knee OA Reduction in Pain on NRS Scale

**Fig. (3).**
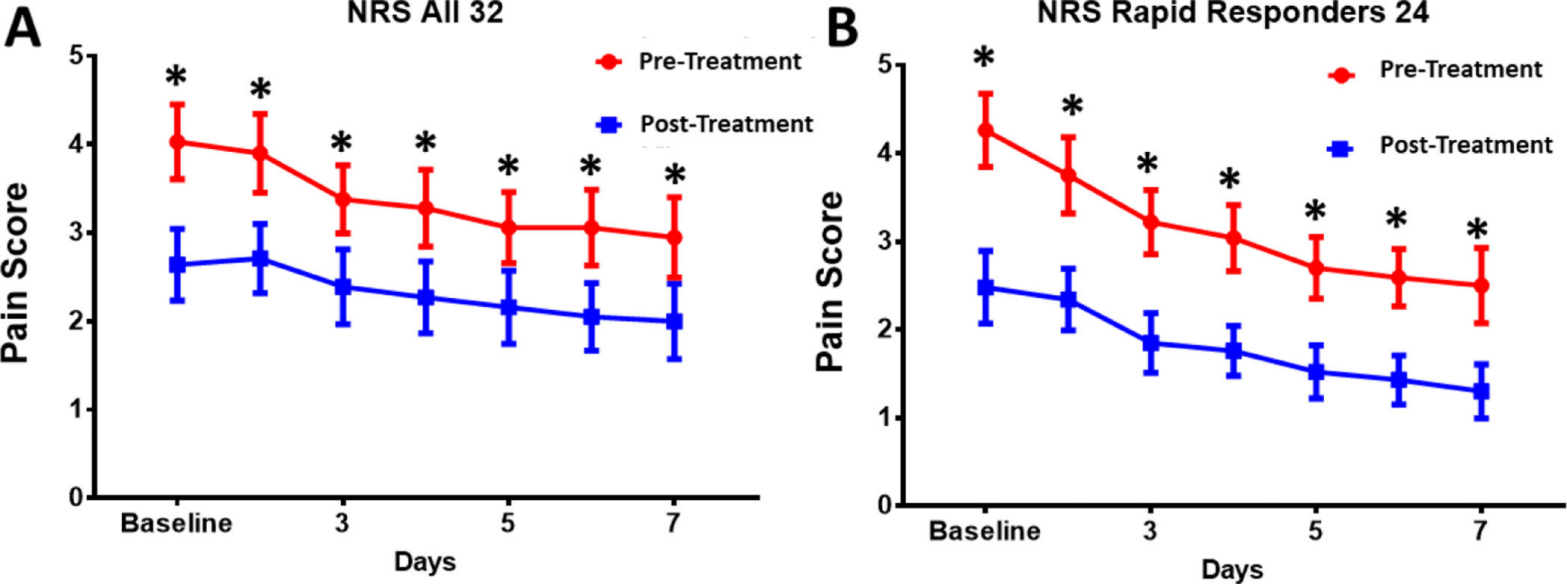
Numeric Rating Score: **A**) “All Subjects n=32” NRS pain showing significant decrease in pain from day 1 through day 7 of treatment. Pain was reduced by 50% from start to end of study (2.06 NRS, p<0.001). **B**) “Rapid Responder n=24” NRS showing significant decrease over 7 days of study. Pain was reduced by 70% from start to end of study (2.96 NRS, p<0.001).

Knee osteoarthritis pain was significantly reduced daily and over the course of the 7-day treatment regimen for both the entire study cohort (100%, n=32) and rapid responders (75%, n=24). For the entire patient cohort, the pretreatment day 1 baseline pain was 4.06 ± 2.39, and after 7 days of treatment, pain decreased to 2.00 ± 2.41, 2.06-point change 7 days (50% decrease, p<0.001, Fig. 3A). The application of SAM with diclofenac patch provided the largest significant daily decrease in pain on the first two days of the study with continued pain reduction thereafter (Fig. 3).

Rapid responders showed a more significant reduction in the NRS pain score. Day 1 baseline pain of 4.26 ± 2.41 to 1.30± 1.5, 2.96-point decrease (70% decrease, p<0.001) over 7 days of treatment (Fig. 3B). Pain reduction significantly decreased daily from baseline day 1 to day 2 to day 3…day 7 (Fig. 3).

### 3.2. Secondary Outcome of Knee Functional Improvement on WOMAC Scale

**Fig. (4).**
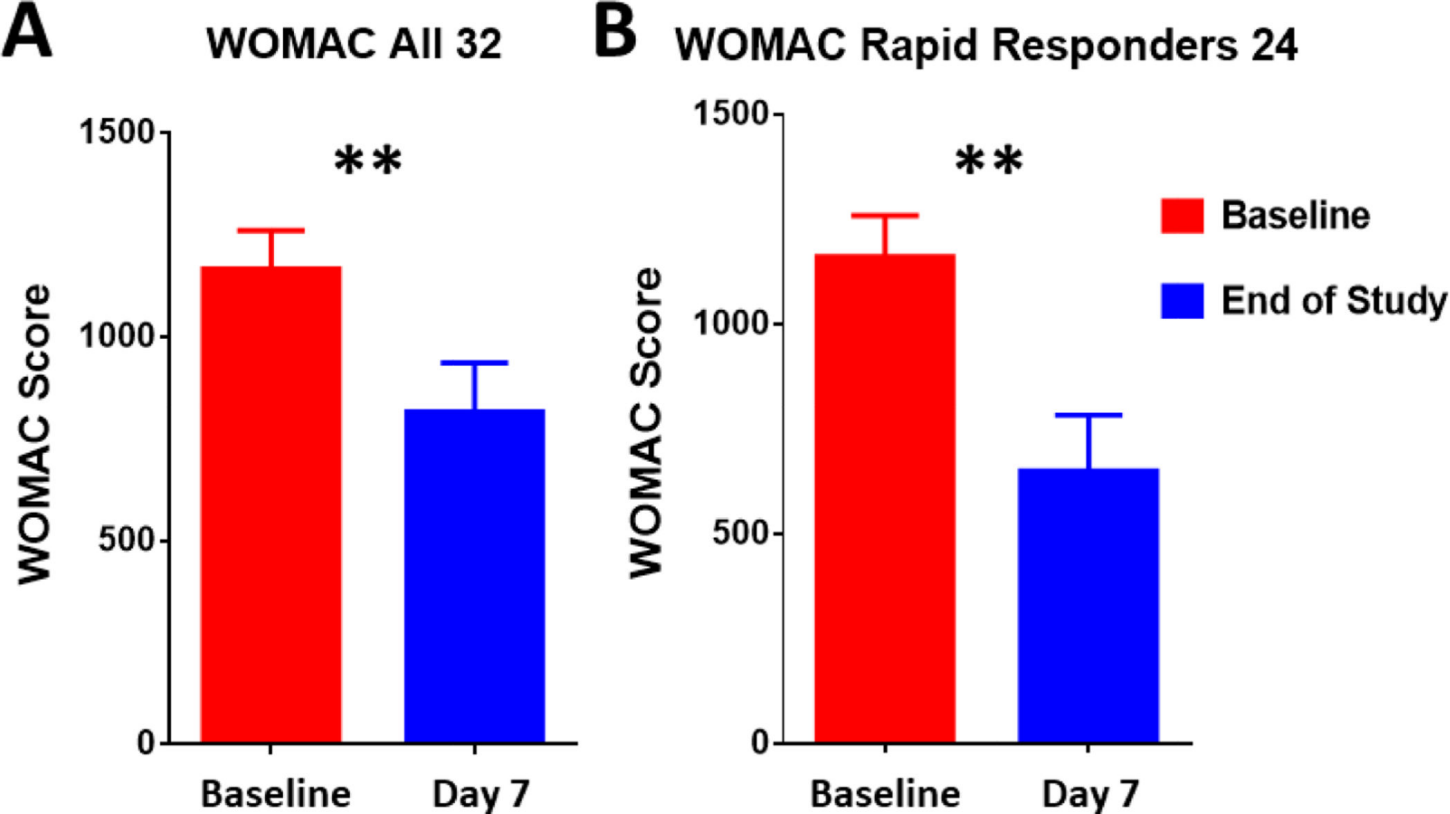
WOMAC Functional Score: **A**) Significant 351-point (p<0.001) decrease in WOMAC score after 7 days of SAM with diclofenac treatment in all subjects n=32. **B**) Rapid Responders n=24 shows more robust 510-point (p<0.001) WOMAC decrease in response to the SAM with diclofenac treatment over 7 days.

The WOMAC score measuring the change in pain, stiffness, and functionality of the knee was significantly improved for SAM with diclofenac patch by 351 points for all subjects (n=32, p<0.001, Fig. 4A), and 510 points for rapid responders (n=24, p<0.001, Fig. 4B). For all subjects, pain score improved by 66-points (p<0.0001), stiffness score improved by 41 points(p<0.001), and functionality score improved by 244 points (p< 0.001) (Table 1). For rapid responders, pain score improved by 92 points (p<0.0001), stiffness score improved by 55 points (p<0.0001), and functionality score improved by 364 points (p<0.0001) (Table 2).

### 3.3. Secondary Outcome of Treatment Usability and Satisfaction

There were no adverse events or study-related complaints across 224 unique treatment sessions. There were no reports of skin burn, skin irritation, or skin sensitization. Over 95% of patients found the device safe, effective, and relatively easy to use and would continue treatment for their knee OA symptoms. By day 7 of the study, 100% of patients in the study reported the device was very easy to use and apply.

## 4. DISCUSSION

Osteoarthritis (OA) is a progressive disease of the joints, resulting in the degradation of cartilage and bone, leading to a reduction of joint space and increased inflammatory response over time [3, 7, 44]. NSAIDs, along with physical therapy, have shown limited efficacy in managing OA associated pain [9, 14, 19, 26, 44, 45]. For many patients, the risks associated with the long-term systemic use of NSAIDs outweigh the modest benefit. While a localized, topical application of NSAIDs is safer for knee OA pain, the efficacy is limited due to drug delivery rates through and across the skin [9, 46]. Diclofenac is one of the more effective and prescribed NSAIDs for OA associated pain [20, 21, 38, 47, 48]. This study used a wearable multi-hour sustained acoustic medicine (SAM) device with 1% diclofenac patch to treat OA associated pain and improve mobility and overall quality of life of patients. SAM applies continuous long-duration ultrasound along with localized delivery of diclofenac to decrease patients’ knee OA pain and improve joint function. In prior preclinical research, SAM has demonstrated the 3.8x increase of diclofenac penetration into and across the skin [49], along with drug delivery enhancement of other drugs over 4-hour treatment protocols [49, 50].

The wearable SAM device and 1% diclofenac ultrasound gel patch could be successfully self-applied by patients for use in the home, providing significant pain relief of KL grade II/III knee OA. No adverse events were noted in the study population, and 94% of participants completed the study. Participants in the study had between a 50% (2.06-point) to a (70%, 2.96-point) pain reduction on the NRS scale. The NRS pain data show an incremental decrease in pain throughout the 7-day treatment with the highest rate of reduction in pain during the first two days. This outcome may be explained by increased penetration of diclofenac into the joint space leading to inhibition of COX1 and COX2 pathways [20, 38], thus relieving the inflammatory response in the joint. Masterson *et al.* (2020) demonstrated SAM increased transdermal diclofenac delivery 3.8 fold greater than topical application alone [51], which were similar to the results of transdermal salicylic acid delivery with SAM by Langer *et al.* (2013) [49]. The sustained treatment and continued pain reduction over the 7-days suggest the potential of lower levels of cytokines to retain pain at a decreased intensity. Functionally, the WOMAC score improved significantly for patients with KL grade II/III knee OA by 351 points for all subjects (n=32, p<0.001) and 510 points for rapid responders (n=24, p<0.001). The improvement in the WOMAC score is indicative of the overall improvement of function and quality of life.

**Fig. (5).**
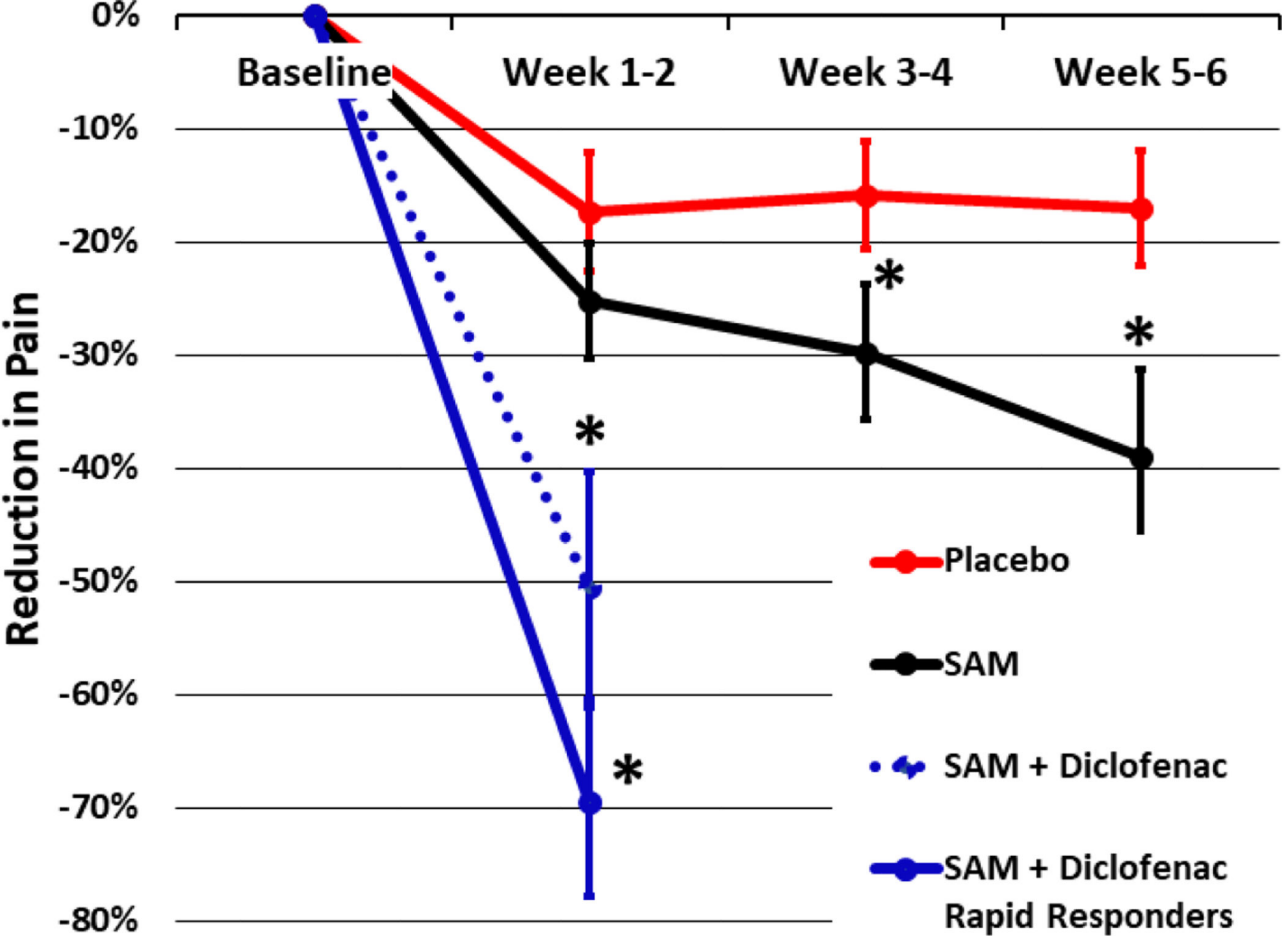
Pain reduction of current study compared with Draper *et al*. (2018). One week of daily SAM with diclofenac patch treatment provides an additional 10% to 30% pain reduction for patients 5 weeks faster than prior reported literature for long-duration ultrasound treatment.

Ultrasound has been shown to be an effective pain management method for arthritis-associated knee pain [29, 37, 45, 52 – 56]. Draper *et al.* (2018) demonstrated a 1.96-point pain reduction on NRS and improvement in total WOMAC score by 505 points comparing the application of SAM active continuous long-duration ultrasound to the placebo group in a 93-subject double-blind, randomized controlled study [33]. This resulted in a 40% knee OA pain reduction for the active treatment group and a 16% pain reduction for the placebo group over 6 weeks of daily treatment. In the current study, we found a 50–70% pain reduction in 1 week of daily SAM with diclofenac patch treatment (Fig. 5). Similarly, Langer *et al.* (2014, 2015) reported a 40% and 48% reduction in knee OA pain after 6 weeks of daily SAM treatment in two pilot case series and one placebo-controlled study, including 66 subjects in total. The current study did not include a placebo group but had similar inclusion criteria, baseline pain, the severity of knee OA (KL grade II/III), and patient demographics. Therefore, the more rapid and robust pain reduction can be attributed to the addition of the 1% diclofenac ultrasound gel coupling patch. Yang *et al*. (2006) has also reported on the efficacy of ultrasound in the reduction of pain with a combination of NSAIDs [39].

Sustained therapeutic ultrasound has also been shown to contribute to improved cartilage thickness. Özgönenel *et al.* (2018) has reported that the application of continuous therapeutic ultrasound in 30 patients showed a significant reduction in visual analog scale (VAS) and WOMAC scores, and improved cartilage thickness in medial femoral condyle after 10 sessions of treatment in a double-blinded trial [31]. Loyola-Sánchez *et al.* (2012) also reported encouraging effects of ultrasound application in managing mild to moderate knee OA in a double-blinded randomized study in 27 patients with a significant increase in cartilage thickness after 20 sessions [57]. Additionally, recent studies and meta-analysis show the efficacy of ultrasound on knee OA pain. Still, the clinical outcome significantly depends on ultrasound parameters such as 1–3MHz frequency, duty cycle, duration, and total energy delivered over treatment time [29, 45, 55].

The FDA approved non-invasive prescription home-use SAM device has little to no adverse effects reported and the contraindications are similar to traditional therapeutic ultrasound [33 – 35, 40, 58]. The SAM device with a diclofenac patch delivers 18,720 Joules of ultrasonic energy and 6g of 1% diclofenac ultrasound gel daily to the arthritic knee of patients. The continuous long-duration ultrasound treatment and locally delivered diclofenac reduce patient pain and improve joint function without requiring systemic NSAID use. The application of SAM to the joint also has diathermic effects [35, 59], which increase the blood flow, oxygenation, and exchange of nutrients. The SAM diclofenac patch ensures ease of patient application of localized diclofenac delivery and a decrease of cytokines such as cyclooxygenase 1 and 2, interleukine 1 β, TNF α, and relative pathways [19, 44, 60 – 65]. The mechanotransductive force of long duration ultrasound along with increased permeability and transport kinetics across the skin leads to increased drug penetration through the skin as well as increased blood flow, which increases the drug penetration into the affected tissue [39, 41, 66 – 69].

Recent preclinical and clinical studies have shown that ultrasound therapy has chondroprotective effects by inhibiting inflammatory factors [36, 52, 64, 70 – 72]. The combined enhanced drug delivery of diclofenac and ultrasound chondroprotective effect makes SAM combined with diclofenac an effective treatment for arthritis associated pain. The current study shows the efficacy of SAM with a 1% diclofenac ultrasound coupling patch in OA patients in a home-use environment, with a significant clinically meaningful decrease in pain and joint stiffness and an increase in functionality and overall quality of life. While the current study did not evaluate the effect of SAM on cartilage, given the short duration of the study, future research is needed to determine the role of sustained, continuous ultrasound as an OA disease-modifying agent.

The SAM device is one of two prescription home-use (1 to 3MHz) ultrasound devices available in the USA. The other device (Exogen®, Bioventus LLC, Durham, NC) was approved by the FDA in the early 1990s for non-thermal application and used a pulsed 20-minute daily ultrasound treatment at a low energy level (140 Joules per 20-minute treatment) [73, 74]. The SAM energy level is approximately 133 times greater and therefore generates significant thermal and non-thermal effects on tissue. The SAM devices used in this study cost $6800, which is significantly less costly than surgery or comorbidities developed from systemic drug use for chronic diseases. SAM treatment should be considered for patients with moderate knee OA pain as a cost conservative treatment option. Future research on long-duration ultrasound as a means for localized drug delivery of NSAID and reduced osteoarthritis disease progression is of great interest. Additional studies could evaluate the dosimetry of SAM and NSAID to provide clinically meaningful pain reduction further while minimizing the use of drugs. The use of SAM with other topical agents such as dexamethasone before, during, and after SAM treatment may also be of interest to the clinical community [75].

## CONCLUSION

Sustained Acoustic Medicine with diclofenac patch rapidly reduced pain and improved joint function in patients with knee osteoarthritis pain. The clinical findings suggest the long-duration continuous ultrasound therapy with diclofenac can provide significant rapid pain reduction for patients with osteoarthritis. The use of SAM device and diclofenac should be considered for patients with moderate pain, requiring a fast-acting intervention in the home setting.

## Figures and Tables

**Table 1.** WOMAC table for all subjects n=32: Shows the baseline score and score after 7 days of treatment. SAM with diclofenac significantly reduced pain and stiffness and improved functionality.

-	Pre-Treatment	Post-Treatment	Mean 95% CI	p value
**WOMAC Pain**	240 ± 110	173 ± 146	−101 to −32	0.0005
**WOMAC Stiffness**	125 ± 42	84 ± 61	−61 to −22	0.0002
**WOMAC Functionality**	803 ± 393	559 ± 481	−356 to −132	0.0001
**WOMAC Total**	1168 ± 528	816 ± 678	−508 to −195	< 0.0001

**Table 2.** WOMAC table for rapid responders n=24: Shows better response to SAM with diclofenac treatment than “All Subjects” with significant decreases in pain and stiffness, and improved functionality over 7 days of treatment.

-	Pre-Treatment	Post-Treatment	Mean 95% CI	p Value
**WOMAC Pain**	235.42 ± 108.33	143.75 ± 140.73	−132.9 to −50.41	0.0005
**WOMAC Stiffness**	124.96 ± 40.38	69.79 ± 60.37	−81.76 to −28.57	< 0.0001
**WOMAC Functionality**	800.22 ± 393.48	436.46 ± 445.05	−557.3 to −170.2	< 0.0001
**WOMAC Total**	1160.59 ± 480.81	650 ± 635.62	−745.2 to −276.0	< 0.0001
